# Novel effect of the high risk-HPV E7 CKII phospho-acceptor site on polarity protein expression

**DOI:** 10.1186/s12885-022-10105-5

**Published:** 2022-09-25

**Authors:** María Paula Dizanzo, Marina Bugnon Valdano, Om Basukala, Lawrence Banks, Daniela Gardiol

**Affiliations:** 1 Instituto de Biología Molecular Y Celular de Rosario-CONICET, Facultad de Ciencias Bioquímicas Y Farmacéuticas, Universidad Nacional de Rosario, Suipacha 590. 2000, Rosario, Argentina; 2 Tumour Virology Laboratory, International Centre for Genetic Engineering and Biotechnology, Trieste, Italy

**Keywords:** HPV E7, CKII, PDZ, DLG1 hScrib

## Abstract

**Background:**

Oncogenic Human Papillomaviruses (HPVs) base their transforming potential on the action of both E6 and E7 viral oncoproteins, which perform cooperative or antagonistic actions and thus interfere with a variety of relevant cellular targets. Among them, the expression of some PDZ-containing polarity proteins, as DLG1 and hScrib, is altered during the HPV life cycle and the consequent malignant transformation. Together with the well-established interference of E6 with PDZ proteins, we have recently shown that E7 viral oncoprotein is also responsible for the changes in abundance and localization of DLG1 observed in HPV-associated lesions. Given that the mechanisms involved remained only partially understood, we here thoroughly analyse the contribution of a crucial E7 post-translational modification: its CKII-dependent phosphorylation. Moreover, we extended our studies to hScrib, in order to investigate possible conserved regulatory events among diverse PDZ targets of HPV.

**Methods:**

We have acutely analysed the expression of DLG1 and hScrib in restrictive conditions for E7 phosphorylation by CKII in epithelial culture cells by western blot and confocal fluorescence microscopy. We made use of genome-edited HPV-positive cells, specific inhibitors of CKII activity and transient expression of the viral oncoproteins, including a mutant version of E7.

**Results:**

We here demonstrate that the functional phosphorylation of E7 oncoprotein by the CKII cellular kinase, a key regulatory event for its activities, is also crucial to counteract the E6-mediated degradation of the PDZ-polarity protein DLG1 and to promote its subcellular redistribution. Moreover, we show that the CKII-dependent phosphorylation of E7 is able to control the expression of another PDZ target of HPV: hScrib. Remarkably, we found this is a shared feature among different oncogenic HPV types, suggesting a common path towards viral pathogenesis.

**Conclusions:**

The present study sheds light into the mechanisms behind the misexpression of PDZ-polarity proteins during HPV infections. Our findings stress the relevance of the CKII-mediated regulation of E7 activities, providing novel insights into the joint action of HPV oncoproteins and further indicating a conserved and most likely crucial mechanism during the viral life cycle and the associated transformation.

**Supplementary Information:**

The online version contains supplementary material available at 10.1186/s12885-022-10105-5.

## Background

Persistent infection with high-risk HPVs, as HPV-16 and HPV-18, is linked to the development of cervical cancer, one of the most frequent cancers in women worldwide, especially in developing countries. Moreover, HPV infections have also been associated with other neoplastic events, such as vulvar, anal, vaginal, penile, and head-and-neck cancers, pointing to HPV as a major infectious carcinogen in humans [1].

The main transforming activities of high-risk HPVs depend on the expression of both E6 and E7 viral oncoproteins. These proteins have the ability to interact and interfere with a large number of key cellular regulators to induce a favourable cellular setting for efficient viral replication [2–6]. Furthermore, the continuous expression of these oncoproteins during persistent HPV infections can lead to cell transformation. Among their strategic functions, E7 proteins stimulate cell cycle progression, at least partly by targeting the pRb pocket protein, while E6 inhibits apoptosis through affecting the p53 oncosuppresor [7]. However, the number of different cellular protein partners and the diverse biological processes targeted by these viral proteins is very large and, in addition, both E6 and E7 can have cooperative or antagonistic activities when subverting a particular cell factor or pathway [6].

Among E6 partners, the intriguing PDZ (PSD95/DLG1/ZO-1) domain-containing proteins have been extensively analysed [8, 9]. The high-risk HPV-derived E6 proteins interact with the PDZ domains via a PDZ binding motif (PBM) located in the C-terminus [8, 10]. The PDZ cellular proteins are molecular scaffolding elements that orchestrate protein complexes involved in the maintenance of cell–cell contacts, cell polarity and cell signalling networks, all processes whose deregulation can be crucial to malignant transformation [9, 11, 12]. Therefore, the association of HPV E6 with PDZ proteins, and the consequences of these interactions regarding PDZ proteins misexpression, have been widely investigated, with a focus on the role of these events in cancer development. Moreover, some studies indicate that the presence of HPV E7, which is expressed together with E6 during HPV-induced tumorigenesis, can impact on the E6-mediated deregulation of PDZ proteins [13, 14].

Discs Large 1 (DLG1) is one of the first and best-characterized PDZ-containing partners of HPV E6, being targeted by almost all E6 proteins from the high-risk HPV types [15, 16]. DLG1, together with the Lethal giant larvae and Scribble proteins, form the conserved Scrib polarity complex. In epithelial cells, DLG1 localizes to the adherens junctions in association with cytoskeleton components, where it coordinates junction formation and stability, contributing to the maintenance of apico-basal polarity as well as participating in transduction signals that control cell proliferation [12, 17, 18]. Furthermore, DLG1 expression was shown to be low or even absent in the later stages of tumour development in different cancers, which strongly suggests that this cellular protein may have oncosuppressor functions [11]. Initially, it was demonstrated that over-expression of high-risk HPV E6 protein can stimulate DLG1 degradation through the ubiquitinylation-proteasomal system [10, 19]. This finding was corroborated by the fact that DLG1 expression is very low in the later stages of cervical cancer development [20, 21], suggesting a role for DLG1 targeting in HPV-induced cell transformation. However, subsequent studies showed significant levels of DLG1 in HPV-positive cervical cancer cell lines, indicating that E6-mediated degradation of DLG1 is incomplete [22]. Moreover, in some squamous intraepithelial precursor lesions (SIL), DLG1 levels are increased in comparison with normal epithelium, although with notable alterations in its cellular distribution [20, 21]. In addition, in organotypic raft cultures expressing HPV-18 E6 (18E6) and E7 (18E7) oncoproteins, high levels and mislocalized expression of DLG1 were observed, in concordance with the data for SIL biopsies [13].

These findings suggest that changes in DLG1 expression could have an impact in HPV pathogenesis, since DLG1 functions are strictly dependent on its correct subcellular distribution [23, 24]. They also indicate that the effects of the HPV-DLG1 association are complex and that their final outcome depends on the biological context during tumour development and in the joint expression of both HPV oncoproteins.

In line with the above, we recently reported that the presence of both HPV-18 oncoproteins in epithelial cells produces a striking mislocalization of DLG1 from the cell borders to the cytoplasm. Moreover, the expression of 18E7 increases DLG1 protein levels, probably by contributing to its stabilization and/or by preventing E6-mediated DLG1 degradation. This suggests that 18E7 protein, when co-expressed with E6, may also have a role in the regulation of DLG1 levels, which would be consistent with the concept that both proteins are complementary in many viral activities [14].

We wanted to pursue these findings to search for possible mechanisms regulating the effects of E7 on DLG1 expression. A key regulatory element of E7 activity is its phosphorylation by Casein Kinase II (CKII) which increases the interaction of E7 with its cellular partners and may thus control different signalling mechanisms [25–28]. In line with this, the E7 phosphorylation by CKII was shown to be crucial for 18E7 activities in maintaining the transformed phenotype [28]. Hence, it resulted interesting to investigate if this post-translational modification was also involved in the E7 effect over DLG1 expression.

Remarkably, human Scribble protein (hScrib), another PDZ-containing protein of the Scrib polarity complex, is likewise a target of high-risk HPV E6 [29]. While being the main target of HPV-16 E6 (16E6) protein for ubiquitin-mediated degradation, it has also been shown to be degraded to a lesser extent by 18E6 [30, 31]. As for DLG1, hScrib levels were found to be increased and mislocalized in some SIL samples, compared with normal tissues [32]. Taking this in mind we also wanted to investigate potentially common mechanisms that regulate diverse PDZ targets during HPV infection and malignant progression.

In summary, in this report we show that E7 phosphorylation by CKII plays an essential role in the ability of this viral protein to regulate the pattern of expression of both DLG1 and hScrib and, moreover, this E7 activity is conserved among different high-risk HPV types. This study provides new information on HPV oncoprotein activities regarding the regulation of PDZ polarity proteins, further indicating their implication in the HPV life cycle and the associated pathogenesis.

## Methods

### Cell culture and transfection

The epithelial cells used were the Human Embryonic Kidney 293 (HEK293, ATCC #CRL-1573), C4-1 (ATCC CRL-1594) and two mutant cell lines (B8 and A15) with a double amino acid substitution S32A/S34A within the CKII phospho-acceptor site of HPV18E7, generated through genome editing, as described by Basukala et al. [28]. B8 and A15 cells were provided by the Tumour Virology Laboratory, International Centre for Genetic Engineering and Biotechnology, Trieste, Italy, where they were created. All cell lines were grown in Dulbecco’s modified Eagle’s medium (DMEM) (Gibco, NY, USA), supplemented with 10% (v/v) fetal bovine serum (Gibco, NY, USA), 300 μg/ml glutamine and 100U/ml penicillin–streptomycin. The cultures were maintained at 37 °C, in a 5% CO_2_ atmosphere.

For cell transfection, the calcium phosphate precipitation method was used, as previously described [10]. Briefly, cells were seeded in appropriate dishes at 50–60% confluence. After 24 h, the transfection solution was prepared with the respective DNA in 100 µl Tris–EDTA (TE) buffer, 11.2 µl 2.5 M CaCl_2_ and 100 µl 2 × HBS, pH 7.12 (50 mM Hepes pH 7, 280 mM NaCl, 1.5 mM Na_2_HPO_4_.7H_2_O). After gentle mixing followed by incubation for 30 min at room temperature, the transfection mixture was added to the desired plate.

### Plasmids

The DNA sequence coding for 18E6 oncoprotein was cloned into pseyfp2-c1 (expression plasmid encoding the super enhanced yellow fluorescent protein 2) and pmTurq1-c2 (encoding an enhanced version of the cyan fluorescent protein) vectors to obtain pseyfp2-18E6 and pmTurq2-18E6. The DNA sequence coding for 16E6 was cloned into pegfp-c1 (expression plasmid encoding the enhanced green fluorescent protein) resulting in pegfp-16E6. The DNA sequence coding for 18E7 oncoprotein was cloned into pegfp-c1 and pLPC-mCherry (expression plasmid encoding the red fluorescent protein) vectors resulting in pegfp-18E7 and pLPC-Cherry-18E7, respectively. The C-terminal FLAG-tagged 18E7 and HPV-16 E7 (16E7) wild type or mutant CMV plasmids were previously described [28].

The DNA sequence coding for hScrib was cloned into HA-tagged pCDNA-3 plasmid, resulting in pCDNA-HA-Scrib. The pegfp-DLG1 and pmTurq2-DLG1 vectors were previously described [14, 33]. A plasmid encoding β-galactoside (β-gal) protein was included in all transfections as a control of equal transfection efficiency.

### Antibodies

Antibodies used were mouse monoclonal anti-HA (12CA5, Sigma Aldrich, St. Louis, MO, USA), mouse monoclonal anti-GFP (B-2, Santa Cruz Biotechnology, Santa Cruz, CA, USA), mouse monoclonal anti-DLG1 (2D11, Santa Cruz Biotechnology, Santa Cruz, CA, USA), mouse monoclonal anti-Scrib (C-6, Santa Cruz Biotechnology, Santa Cruz, CA, USA), mouse monoclonal anti-FLAG-M2-peroxidase (Sigma Aldrich, St. Louis, MO, USA), mouse monoclonal anti-18E6 (BF7, Santa Cruz Biotechnology, Santa Cruz, CA, USA), rabbit monoclonal anti-CDK2 (D12, Santa Cruz Biotechnology, Santa Cruz, CA, USA), mouse monoclonal anti- α tubulin (GTU-88, Sigma Aldrich, St. Louis, MO, USA) and mouse monoclonal anti- β-Gal (Promega, Madison, USA). Secondary anti-rabbit HRP and anti-mouse HRP antibodies were obtained from Dako.

### Cell protein extracts and Western blot

Whole-cell extracts were prepared by resuspending cells directly in SDS-sample buffer (125 mM Tris–HCl pH 6.8, 2% SDS, 20% glycerol, 0.01% bromophenol blue and 10% b-mercaptoethanol). When specified, cells were treated with 2.5 μM Cysteine Kinase II (CK II) inhibitor (Silmitasertib, CX-4945, MedChem) for 4 h prior to protein extraction.

Equal amounts of protein extracts were separated by SDS-PAGE and transferred onto 0.22 µm nitrocellulose membranes. Membranes were blocked in 5% non-fat dry milk in TBST (20 mM Tris–HCl pH 7.5, 150 mM NaCl, 0.1% Tween-20) for 1 h and probed with the appropriate primary and secondary antibodies. The blots were then developed using the ECL Western blotting detection reagent (GE Healthcare, Chicago, Illinois, USA) according to the manufacturer’s instructions. Blots were cut prior to hybridisation with the indicated antibodies, hence, the original images of full-length membranes cannot be provided. The original unprocessed used blots with visible membrane edges have been included in Fig. S3 to Fig. S9. The relevant bands showed in the manuscript figures are indicated by a dashed lined rectangle.

Protein band intensities were quantified using the FIJI software [34]. In order to test the statistical significance of protein variations ANOVA-test and multivariate Tukey analysis were performed.

### Fluorescence microscopy and immunofluorescence

HEK293 cells were grown on glass coverslips (350,000 cells/35 mm diameter dish), transfected for 24 h. When specified, cells were treated with 2.5 μM Cysteine Kinase II (CK II) inhibitor (Silmitasertib, CX-4945, MedChem) for 4 h prior to fixing. The cells were fixed using 4% paraformaldehyde in phosphate-buffered saline (PBS) for 15 min at room temperature.

Cell cultures overexpressing fluorescent fusion proteins were directly mounted with SlowFade reagent (Molecular Probes, Thermo Fisher Scientific, USA). Fluorescence microscopy images were collected with a Carl Zeiss LSM880 confocal microscope following the sequential acquisition mode (Carl Zeiss, Germany). A 63 × NA 1.4 plan apochromat oil immersion objective was employed. The quantification analysis of DLG1 levels in cells borders was performed using the FIJI v1.52 software [34], following a previously described algorithm. Briefly, the segmentation plugin “Trainable Weka Segmentation v3.2.34”, a classification tool based on machine learning [35], was applied on each image to create a template that would automatically find the labelled cellular proteins associated to the cell borders by providing examples of membrane and cytoplasmic fluorescence signal. Each segmented image was converted into a binary mask and multiplied by the corresponding original image to obtain the final “classified image”. Therefore, these images conserved the original value of the pixels associated with the border fluorescence signal, while assign a value of 0 to each pixel associated with the cytoplasm. In order to process automatically the input fluorescence images, an “in-house” macro IJM code was written. To assess the fluorescence intensity in individual cells, regions of interest (ROIs) were manually outlined along cell perimeters to include the full membrane width and thickness using the original images.

On each original and classified image, DLG1 fluorescence was quantified as the sum of pixel intensities, expressed as raw integrated density (RawIntDen) per ROI. Therefore, the RawIntDen and the area of each ROI for the total pixels (original image) and only for the membrane pixels (classified image) were measured.

In order to automatically analyze the fluorescence images with high reproducibility and precision, an in-house macro code was written in “Image J Macro” (IJM) language. Kruskal–Wallis test followed by Dunn's comparisons (*p* value < 0.01) was performed.

## Results

### Mutation of the HPV-18 E7 CKII phospho-acceptor site prevents the E7-mediated stabilization of DLG1

In order to clarify the mechanisms involved in the regulation of the expression of DLG1 PDZ-protein by HPV oncoproteins, we first investigated the relevance of the CKII-dependent E7’s phospho-acceptor site.

**Fig. 1:**
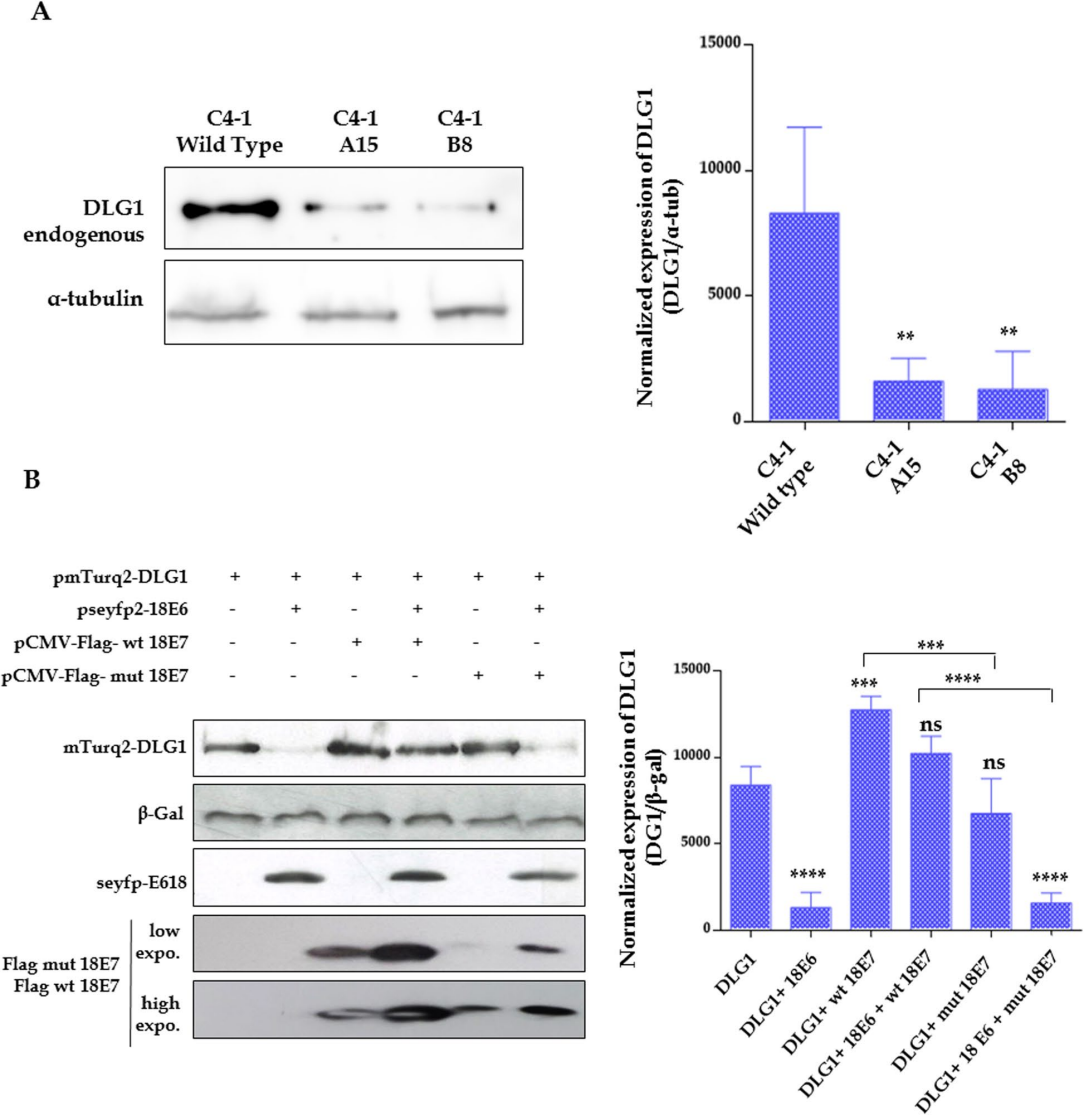
Mutation of the HPV-18 E7 CKII phospho-acceptor site prevents the stabilization of DLG1. **A** C4-1 wild type cells and the CKII HPV-18 E7 phospho-acceptor mutant derivative (C4-1 B8 and C4-1 A15) cells were grown for 24 h and whole protein extracts were analysed by Western Blot (Left panel). The endogenous expression of DLG1 was ascertained using the specific anti-DLG1 antibody. α-tubulin was used as loading control. Right panel, densitometry analysis of western blots for DLG1 expression, normalised for α-tubulin levels in each corresponding different condition (mean ± SD, *n* = 3). Asterisks denote significant differences determined by ANOVA test (**p < 0.05). **B** HEK293 epithelial cells were transiently transfected with 5 µg of pseyfp2-18 E6, 5 µg of wild type or 5 µg of mutant pCMV-Flag-18 E7 and 1 µg of pmTurq2-DLG1 plasmid. Cells were harvested 24 h post-transfection and the expression of fusion proteins was assessed by Western Blot (Left panel) using an anti-Flag (wild type and mutant 18E7), anti-18E6 and anti-GFP, which recognizes mTurq2-DLG1. β-Galactoside levels were used as transfection control. For the assessment of the E7-Flag proteins two images with different degree of exposition are shown for a better appreciation. Right panel**,** densitometry analysis of western blots for DLG1 expression for equal β-galactoside levels in each corresponding different condition (mean ± SD, *n* = 3). Asterisks denote significant difference determined by ANOVA test and a Multiple Comparisons Tukey´s test (***p < 0.005, ****p < 0.0005) and “ns” indicates no significant changes. Original blots are presented in the Fig. S3 and Fig. S4

For this, we analysed DLG1 levels in the cervical carcinoma C4-1 cells, which bear a single copy of the HPV-18 genome, and in two derived cell lines (B8 and A15), that were generated by genome editing, introducing mutations into the 18E7 CKII site which prevent its phosphorylation [28]. DLG1 abundance was ascertained by Western blot using total protein extracts from C4-1 and the two mutant derivative lines. As can be seen in Fig. 1A, DLG1 levels in C4-1 cells are significantly higher than in either B8 or A15 cells. As the genetic difference between these cell lines lies only in the E7 CKII site, these results point towards the possibility that only the wild type E7 protein expressed in C4-1 cells can rescue DLG1 from E6-mediated degradation, as previously reported [14]. Since mutations preventing E7 phosphorylation by CKII can circumvent this rescue, altogether these data suggest the relevance of this site in DLG1 stabilization by E7.

To further investigate these findings, we performed transient transfection experiments. HEK293 cells were co-transfected with plasmids expressing DLG1, 18E6 and wild type or CKII mutant 18E7, as indicated in Fig. 1B. After 24 h, cells were harvested and the levels of DLG1 were ascertained by Western blot. The results indicate that, as expected, the levels of DLG1 are greatly reduced in the presence of 18E6, as a result of its E6-mediated degradation [10]. However, DLG1 levels are not diminished in the presence of wild type E7 protein, even when E6 is expressed, in agreement with our previous studies [13, 14]. Remarkably, no rescue of DLG1 levels was observed in the presence of the CKII E7 mutant (Fig. 1B), in line with the data shown in Fig. 1A. Moreover, as we reported [14], wild type E7 alone can increase DLG1 levels, even in the absence of E6; nevertheless, no significant changes in DLG1 abundance were detected when expressing the mutant version of E7 in the absence of E6 (Fig. 1B). Together, these results suggest that the regulation of DLG1 abundance by E7 depends on the presence of an intact CKII phospho-acceptor site in the viral protein.

### The CKII phosphorylation of 18E7 is involved in stabilizing DLG1

**Fig. 2:**
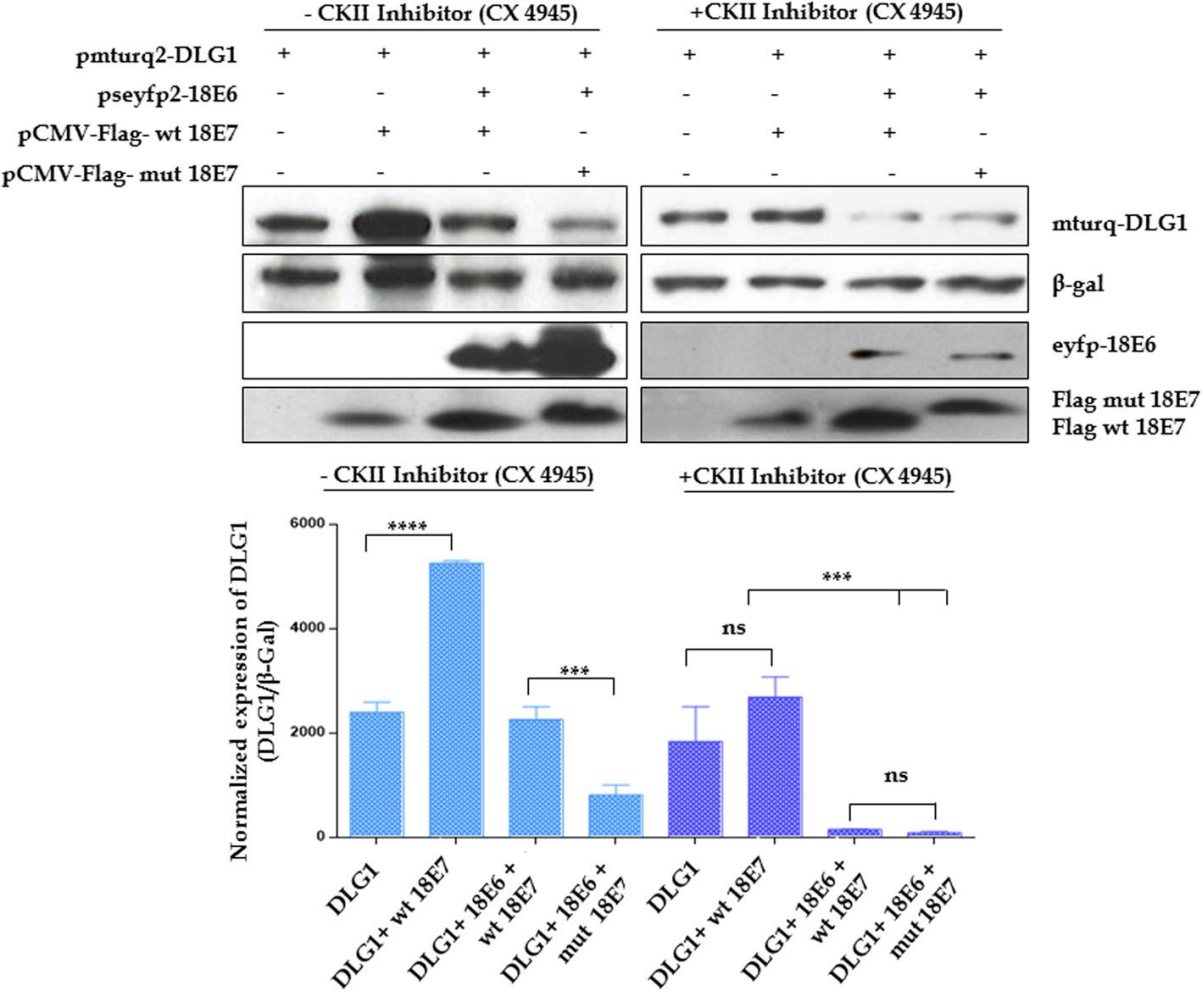
Phosphorylation of 18E7 by CKII is required for stabilization of DLG1 levels. HEK293 cells were transfected with 1 μg of pmTurq2-DLG1, 5 μg of pseyfp-18E6 and/or 5 μg of wild type or mutant pCMV-Flag-18 E7, as indicated. Fusion proteins were assessed by western blot 24 hs post transfection (Upper panel) using an anti-Flag (E7), anti-18E6 and anti-GFP, which recognizes mTurq2-DLG1. β-Galactoside levels were used as transfection control. When indicated, CX-4945 CKII inhibitor was added to the cells 4 h before harvesting. Images with different exposure levels area shown in Fig. S5. Bottom panel, densitometry analysis of DLG1 levels normalized to β-Galactoside expression. Asterisks denote the significant difference between conditions tested by ANOVA test and a Multiple Comparison Tukey´s test (***p < 0.005, ****p < 0.0005) and “ns” indicates no significant changes. The results are representative of three independent experiments. Original blots are presented in Fig. S5

Next, we wanted to specifically analyze if CKII phosphorylation of 18E7 is required for E7's regulation of DLG1 levels. HEK293 cells were co-transfected with plasmids expressing DLG1, 18E6 and wild type or mutant 18E7, and then treated with the specific CKII inhibitor CX-4945 [36] for four hours before protein extraction. The levels of DLG1 were ascertained by Western blot both for inhibitor-treated and untreated (control) cells and the results are shown in Fig. 2. Interestingly, the addition of CKII inhibitor abolishes the ability of wild type E7 to significantly increase the amount of DLG1. Moreover, in cells co-transfected with E6 and wild type E7, CX-4945 treatment reduces DLG1 to levels similar to those seen for cells co-transfected with CKII E7 mutant, indicating that the specific phosphorylation inhibitor also prevents the E7-mediated rescue of DLG1 from E6 degradation (Fig. 2, right panel). These observations suggest than an active CKII-mediated phosphorylation of HPV E7 is required for the stabilization of DLG1 protein.

Furthermore, DLG1 expression was previously shown to change during the cell cycle [37] and one of the major functions of E7 is precisely the stimulation of cell cycle progression to S phase in a way most likely dependent on CKII phosphorylation [38, 39]. In order to evaluate whether this E7 activity could be involved in changing DLG1 expression we assessed the expression of the cell cycle-dependent kinase -2 (CDK-2), which was also shown to phosphorylate and stabilize DLG1 [37]. As can be seen in Fig. S1 A (uncropped Fig. S8), treatment with CKII inhibitor induces a reduction in both DLG1 and CDK-2 levels in the HPV-positive C4-1 cells and, furthermore, the expression of wild type 18E7 but not of the CKII mutant 18E7 significantly increases the levels of this cell cycle kinase (Fig. S1 B, uncropped Fig. S9). Altogether, these results suggest that the expression of E7 may induce CDK-2, in a CKII dependent manner, which next contributes stabilizing DLG1 in the presence of the HPV oncoproteins.

### The phosphorylation of 18E7 by CKII induces subcellular redistribution of DLG1

**Fig. 3:**
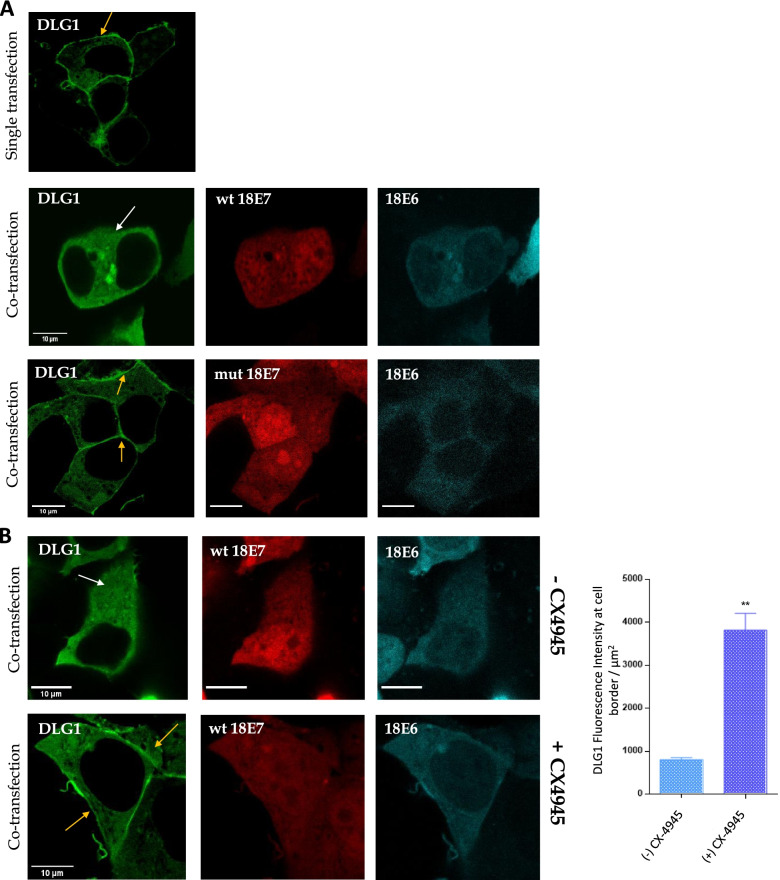
Analysis of the effect of CKII phosphorylation upon DLG1 subcellular localization**. A** An intact E7 CKII phospho-acceptor site is required for DLG1 misdistribution in the presence of both HPV oncoproteins. Upper panel**,** the vector encoding egfp-DLG1 (1.5 μg) was transfected into HEK293 cells and DLG1 localization was analyzed by confocal microscopy (yellow arrow). Middle and bottom panel, the vector encoding mTurq2-18E6 (cyan 0.75 μg), egfp-DLG1 (green 0.75 μg) and wild type or mutant Cherry-18E7 (red 0.75 μg) were co-transfected into HEK293 cells and the localization of each fusion protein was analyzed by confocal microscopy after 24 post transfection, white and yellow arrows indicate DLG1 subcellular localization. **B** Effect of specific CKII inhibitor CX-4549 upon DLG1 localization. HEK293 cells were transfected with pegfp-DLG1 (green 1.5 μg), pmTurq2-18E6 (cyan 0.75 μg) and wild type pCherry-18 E7 (red 0.75 μg) for 24 hs. CX-4945 CKII inhibitor was added to the cells 4 h before fixing, as indicated (+ CX-4945). The bar graph shows the quantification of the DLG1 levels at cell borders in the presence or absence of the inhibitor CX-4945, at least 80 cells were analyzed per condition. All scale bars represent 10 μm. The results are representative of at least 3 independent experiments

To further investigate the contribution of the 18E7 CKII phospho-acceptor site in the regulation of DLG1 expression, we evaluated by confocal fluorescence microscopy assays the cellular localization of this PDZ polarity protein in the presence of 18E6 and the wild type or CKII-mutant 18E7, as indicated in Fig. 3A.

First, the subcellular distribution of DLG1 was examined by transfecting the corresponding pegpf-DLG1 (green) expression vector into HEK293 cells and was found to be primarily located at the cell borders (Fig. 3A, upper panel, yellow arrow), as previously established [40]. Alternatively, HEK293 cells were further co-transfected with the expression vectors pegpf-DLG1 (green), pmTurq-18E6 (cyan) and pLPC-Cherry-wild type or mutant 18E7 (red), using high levels of ectopic DLG1 in order to overcome the E6-mediated degradation and therefore enabling the analysis of protein localization [14]. DLG1 redistribution to the cytoplasmic region in the presence of both 18E6 and wild type 18E7 was observed (Fig. 3A, middle panels, white arrow) as we have previously demonstrated [14]. The control patterns of DLG1 distribution when this protein is independently co-expressed with E6 or E7 is provided in Fig. S2. Also as expected, DLG1 over expression promotes the redistributions of E6 from the nucleus, without a marked mislocalization of DLG1. In the presence of E7, which regularly localizes both in nucleus and cytoplasm, only a minor change in DLG1 expression was observed, in agreement with our previous publication [14] and endorsing the issue that both viral proteins are required for mislocalizing DLG1. However, when the CKII mutant 18E7 was expressed instead of the wild type viral protein, DLG1 protein was retained at the cell–cell contacts (Fig. 3A, bottom panel, yellow arrow), indicating a role for the CKII-dependent E7 phosphorylation in DLG1 regulation.

To further investigate the effect of E7 CKII phosphorylation upon the localization of DLG1, we performed transient transfection experiments as described above, but in the presence of CX-4945, a CKII inhibitor. HEK293 cells were co-transfected with the fluorescent protein vectors indicated in Fig. 3B and were incubated with or without CX-4945 for four hours. The cells were then fixed and the subcellular localization of each protein was determined by confocal microscopy. Again, in the absence of the inhibitor, we detected the redistribution of DLG1 from the cell borders to the cytoplasm when E6 and wild type E7 are co-expressed (Fig. 3B, upper panel, white arrow). However, DLG1 mislocalization is much less evident when the transfected cells are treated with CX-4945, since a notable amount of DLG1 protein is retained at the cell contacts (Fig. 3B, bottom panel, yellow arrows). In order to determine the proportion of mislocalized DLG1 in both conditions, we carried out a quantitative analysis using the FIJI v1.52 software. Figure 3B (right panel) shows DLG1 fluorescence intensity at the cell borders in each experimental setting and the results indicate that, in the presence of CKII inhibitor, DLG1 levels at the cell-to-cell contacts are significantly higher than in the control.

All together, these data indicate that an intact phospho-acceptor site on 18E7 and the active CKII phosphorylation of 18E7 are required for the observed redistribution of the PDZ protein in the presence of both E6 and E7 HPV oncoproteins.

### HPV E7 protein regulates the expression of other HPV E6 PDZ targets

**Fig. 4:**
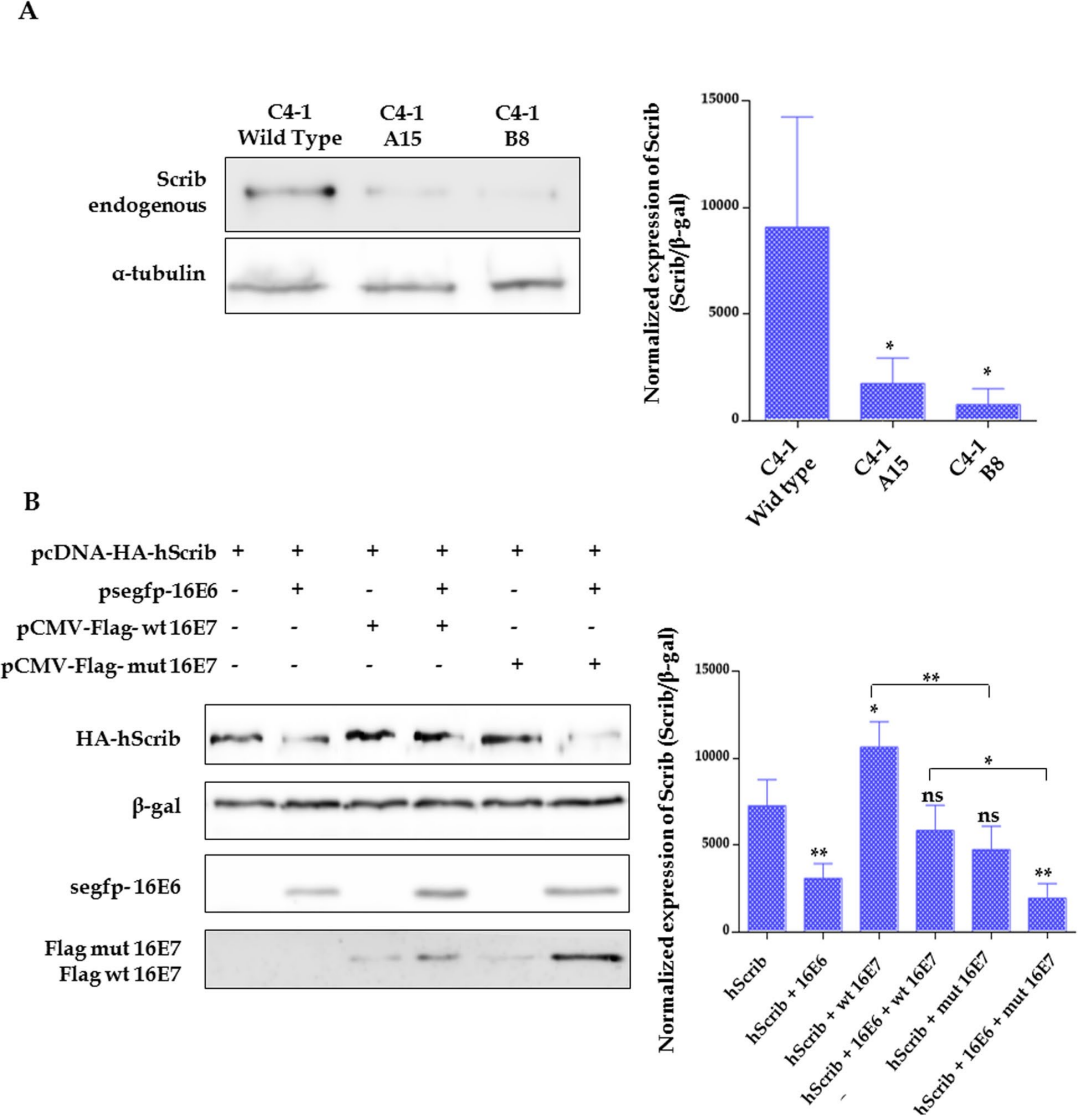
HPV E7 can stabilize the different PDZ targets of E6 and this activity is conserved between the E7 proteins from several high-risk HPVs**. A** C4-1 wild type cells and the CKII HPV-18 E7 mutated cell lines (C4-1 B8 and C4-1 A15) were grown for 24 h and then whole protein extracts were analysed by Western Blot (Left panel). The endogenous expression of hScrib was then studied using a specific mouse-anti-Scrib antibody. Original blots are presented in Fig. S6. Right panel, densitometry analysis of Western blots for hScrib expression for equivalent α-tubulin levels in each corresponding different condition (mean ± SD, *n* = 3). Asterisks denote significant difference determined by ANOVA test (*p < 0.05). **B** HEK293 epithelial cells were transiently transfected with 2.5 µg of pegfp-16 E6, 2.5 µg of wild type or mutant pCMV-Flag-16 E7 and 0.5 µg of pcDNA-HA-Scrib plasmid. Cells were harvested 24 h post-transfection and the expression of fusion proteins was assessed by Western Blot (Left panel) using an anti-Flag (E7), anti-GFP (E6) and anti-HA (hScrib). β-Galactoside levels were used as transfection control. Images with different exposure levels and original blots are presented in Fig. S7. Right panel**,** densitometry analysis of western blots for hScrib expression for equal β-galactoside levels in each corresponding different condition (mean ± SD, *n* = 3). Asterisks denote significant difference determined by ANOVA test and a Multiple Comparisons Tukey´s test (**p < 0.05, ***p < 0.005) and “ns” indicates no significant changes

Next, we wanted to extend our analysis to other PDZ proteins known to be targets of high-risk HPV E6 oncoproteins, such as hScrib [29]. Hence, we first evaluated whether the mutations in the CKII phosphorylation site of 18E7 could affect the hScrib protein levels. For this, we analysed by Western blot the C4-1 parental cells and the mutated cell lines (B8 and A15), previously described (this report and [28]). Interestingly, as shown in Fig. 4A, both 18E7 mutant cell lines have lower levels of hScrib than the wild type cell line. These data, similar to our previous results on DLG1, indicate a role for 18E7 in the rescue of hScrib from E6 degradation, while suggesting that this would depend on its phosphorylation by CKII.

To further investigate these findings, and considering that the PDZ protein hScrib is preferentially targeted by 16E6, we performed a series of transient transfection experiments using HPV-16 proteins. HEK293 cells were co-transfected as indicated in Fig. 4B and harvested after 24 h to ascertain the levels of hScrib by Western blot. As expected, the abundance of hScrib was greatly reduced in the presence of 16E6, most likely due to its E6-mediated degradation [29]. However, hScrib levels were rescued by the co-expression of wild type, but not CKII mutant, HPV-16 E7 protein (Fig. 4B). Moreover, as was demonstrated for 18E7 and DLG1, we observed that wild type 16E7 can increase the expression of hScrib, even in the absence of E6. Conversely, the CKII mutant of 16E7 did not induce any statistically significant changes in hScrib abundance (Fig. 4B). Overall, these results indicate the stabilization of another PDZ target, hScrib, by CKII-phosphorylated 16E7.

## Discussion

Although HPV infections are an important issue for public health, many of the mechanisms involved in the viral life cycle and pathogenesis are elusive. Important questions remain about the complex interplay between key cellular target proteins and the E6 and E7 viral oncoproteins, whose co-expression is required to create an environment compatible with the viral life cycle and which, concomitantly, can drive malignant progression. With no known enzymatic activities, these viral proteins carry out their actions by perturbing a plethora of different cellular factors, including several PDZ polarity proteins ([6] for a review). In this report we provide new insights on the regulation of PDZ proteins by HPV.

Despite the well-known joint action of E6 and E7, it is not always clear when these oncoproteins perform cooperatively or, instead, when they have opposed outcomes; and this is also true for their effect on PDZ proteins. While high-risk HPV E6's detrimental impact on several of these cellular targets is well established [8, 41], much less is understood about the role of E7 oncoproteins in their regulation. In this regard, we have recently reported that the combined expression of both HPV-18 major oncoproteins promotes not only the upregulation, but also the subcellular redistribution, of the PDZ cellular protein DLG1 [13, 14]. Moreover, this expression pattern was the same as that observed in HPV-associated cervical lesions, where the mislocalization of DLG1 could have acquired oncogenic traits, contributing to cancer [20, 21].

In this study we have begun to address the mechanisms behind these observations. We here demonstrate the implications of a key modification of E7: its main CKII-dependent phosphorylation. First, using HPV-18-positive cervical cancer cells, edited in the CKII phospho-acceptor site of E7, we found that the genetic mutation of this site is linked to less abundant endogenous DLG1. Moreover, by evaluating the transient expression of this PDZ protein in the presence of wild-type E7 or E7 mutated to be CKII phosphorylation-resistant, individually or together with 18E6, we find that the integrity of the CKII phospho-acceptor site in E7 is required for DLG1 stabilization. It is important to emphasize that, while E7 is known to be regulated by several different post-translational events ([42] for a review), the CKII-mediated phosphorylation of 16E7 has been shown to be crucial for the viral life cycle [43], most likely by favouring the recognition of important cellular targets, including pRb, the pocket protein p130 and the TATA box binding protein (TBP) [39, 44, 45]. In contrast to these direct substrates, HPV-E7 is unable to bind to PDZ-containing proteins [10, 14]. Thus, the impact of E7 on DLG1 levels would be almost certainly due to indirect mechanisms. Based on our current data, we propose that E7's phosphorylation by CKII is a crucial part of the pathways involved. Interestingly, numerous key roles have been established for this phosphorylation in modulating E7's transforming potential [25, 46, 47] and its activities that favour cellular proliferation and invasiveness [28]. In this regard, even though DLG1 was initially identified as a tumour suppressor, it has been paradoxically shown to have pro-oncogenic functions when mislocalized [24, 48] and has been observed to accumulate in the cytoplasm of cancer cells from various different tumours [11, 12]. Hence, in order to favour virus cycle progression, it appears that phosphorylated E7 may promote the accumulation of DLG1, to promote cell growth and proliferation. In line with this, it is important to consider that the cell lines bearing E7 variants unfitted for CKII-mediated phosphorylation were previously shown to present a deficiency in growth rate [28].

It is also worth noting that when E7 is involved in the induction of S-phase through a CKII-dependent mechanism [38, 39, 49], several other relevant cellular kinases are known to be triggered or transcriptionally activated during this process. DLG1 has been shown to be regulated by a complex net of cellular kinases, including the cell cycle regulators CDK-1 and CDK-2 [37, 50]. DLG1 cell distribution and stability are modulated through changes in its phosphorylation status, and specifically some phosphorylated forms of DLG1 have been shown to be less prone to degradation, most likely by being less susceptible to ubiquitination [37]. We observed that CDK-2 levels are reduced by CKII inhibitors and on the other hand, are increased in the presence of wild type 18E7. These data point towards a probable link between cellular kinases expressed during the cell cycle progression activated by E7, in a CKII-dependent way, and more stable forms of DLG1.

Overall, our present results show that when expressing both main HPV oncoproteins, as occurs during the HPV life-cycle, and in HPV-induced tumourigenesis, not only does 18E7 facilitate the cytoplasmic accumulation of DLG1, but also that this effect is mediated by E7 CKII-dependent phosphorylation. Interestingly, an 16E7 variant with an additional CKII-dependent phosphorylation site has been shown to exert increased transforming activity [45], highlighting the relevance of phosphorylation by this kinase in HPV-associated lesions. However, their precise consequences in the viral life cycle or during the different phases towards malignant progression remain to be elucidated. Although CKII is not considered an oncoprotein, studies have shown that its dysregulation can contribute to its oncogenic potential [47, 51]. Moreover, high CKII levels are associated with cell proliferation: it has been found to be greatly increased during malignant transformation in a variety of tumours, raising the possibility of its having a key role in the tumoural phenotype [47, 51–54]. Targeting CKII with specific inhibitors has shown considerable therapeutic potential, and some inhibitors are currently in clinical trials with apparently promising outcomes [42, 55–57]. Indeed, our results using one of these compounds, the CX-4945 molecule, show that selective inhibition of CKII can block 18E7's effect on DLG1, with respect both to its abundance and its subcellular distribution. Thus, we demonstrate that CKII-dependent E7 phosphorylation modulates the cellular levels of DLG1, reinforcing the relevance of HPV E7 modification by CKII to the functionality of this oncogenic viral protein.

Furthermore, our findings indicate that the effects of 18E7 on DLG1 may be common to other polarity proteins, since similar effects are seen with hScrib [58]. hScrib expression is regulated by a complex interplay of different postransductional modifications and, importantly, its overexpression and accumulation in the cytoplasm is associated with malignant progression in various tumours, including cervical cancer [59, 60]. Our data point towards E7 contributing to the stabilization of hScrib and, moreover, put forward that this regulation depends on CKII. In addition, given that 16E7 also appears to function similarly to 18E7, our results suggest that this mechanism is conserved among different high-risk HPVs [30, 31].

Thus, the efficient phosphorylation of E7 by CKII could have direct consequences upon its partner recognition and, given the multiplicity and variety of E7 targets, numerous signalling pathways may be controlled by this phosphorylation. These pathways then might regulate PDZ protein expression to favour the accumulation of the subcellular fractions most resistant to E6-mediated degradation. It is important to remark that both DLG1 and hScrib are highly expressed in cervical low-grade SILs (LSILs), with a distribution pattern similar to that described here, when HPV E6 and phospho-E7 are co-expressed. In these lesions HPVs are replication-competent, therefore, it can be speculated that differential expression of the PDZ proteins could be relevant for the virus life cycle. However, we have previously shown that those LSILs with the highest DLG1 levels are prone to progress to more severe lesions, compared with those LSILs with lower DLG1 abundance. As progression to high-grade lesions is detrimental to virus replication, there may be a tight balance between the antagonistic activities of HPV E6 and E7 regarding the regulation of the PDZ proteins, an interesting issue that deserves further investigation. Although the exact downstream effects of E7's CKII phosphorylation remain to be unravelled, we have started to elucidate the events involved in the E7-dependent modulation of different PDZ polarity targets, unveiling a regulatory process conserved among different high-risk HPVs.

## Conclusions

In conclusion, the co-expression of both E6 and E7 HPV oncoproteins induced changes in the expression of the DLG1 and hScrib PDZ proteins and, interestingly, this effect depends on the phosphorylation of E7 by CKII. This finding underlines a novel role for CKII in regulating E7 activities linked to the misexpression of key polarity proteins. Remarkably, this E7 activity is conserved among different high-risk HPV types suggesting common strategies conducting to loss of cell polarity during HPV carcinogenesis. This study contributes to explain potential mechanisms behind the observed differential expression of these PDZ proteins during the malignant progression associated to the viral infection. Nevertheless, future research is still needed to completely understand how, when and why the HPV oncoproteins target the PDZ proteins during the virus life cycle and pathogenesis.

## Supplementary Information

**Additional file 1: Fig. S1.** A. CKII inhibitor induces a reduction in DLG1 and CDK-2 levels in HPV-positive cells. **Fig. S1.** B. HPV-18 E7 protein increases the levels of CDK-2 in a CKII-dependent manner. **Fig. S2.** Analysis of DLG1 expression in the presence of either E618 or E718. Upper panel. **Figure Supplementary 3.** UncroppedFigure 1 A. **Figure Supplementary 4.** Uncropped Figure 1 B. **Figure Supplementary 5.** Uncropped **Figure 2. Figure Supplementary 6.** UncroppedFigure  4 A. **Figure Supplementary 7. **Uncropped Figure 4 B. **Figure Supplementary 8.** Uncropped Figure Supplementary 1. **Figure Supplementary 9. **Uncropped Figure Supplementary 1 B.

## Data Availability

The raw data supporting our findings can be found in Fig. S3 to Fig. S9 or can be requested to the authors.

## Abbreviations

CDK-2: Cyclin dependent kinases 2
CKII: Casein kinase II
DLG1: Human disc large 1
EGFP: Enhanced green fluorescent protein
HA: Influenza virus hemagglutinin epitope
HPV: Human papillomavirus
hScrib: Human scribble protein
HSIL: High-grade SIL
LSIL: Low-grade SIL
mCherry: Cherry fluorescent protein
mTurq2: Enhanced version of cyan fluorescent protein
PBM: PDZ-binding motif
PDZ: PSD-95/DLG1/ZO -1 domains
seyfp2: Super enhanced yellow fluorescent protein
SIL: Squamous Intraepithelial precursor Lesions
