# Supplementary material for: Novel effect of the high risk-HPV E7 CKII phospho-acceptor site on polarity protein expression
Source: BMC Cancer. 2022 Sep 25;22:1015. doi: 10.1186/s12885-022-10105-5 (PMC9509620; doi:10.1186/s12885-022-10105-5)

Figure Supplementary 1.

A

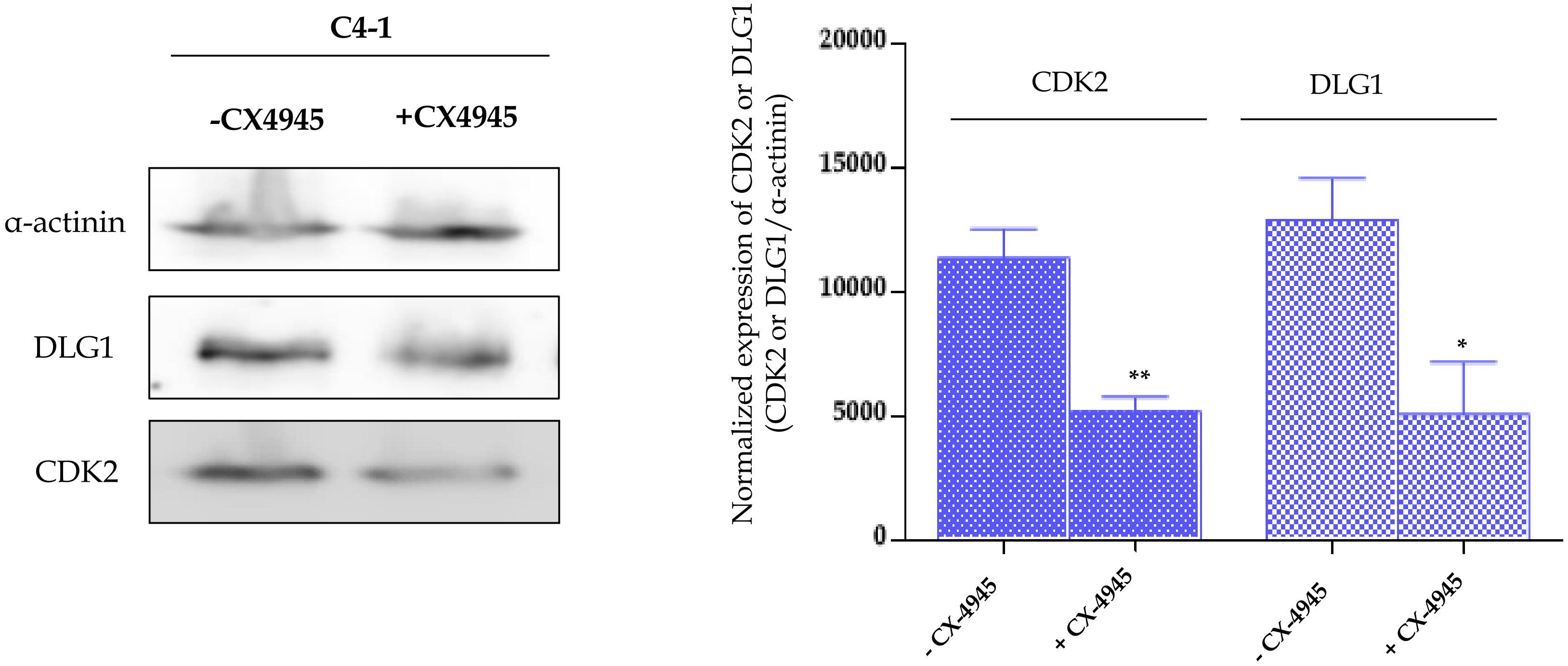

Fig. S1. A. CKII inhibitor induces a reduction in DLG1 and CDK-2 levels in HPV-positive cells. C4-1 wild type cells were grown for 24h and whole protein extracts were analysed by Western Blot (**Left panel**). The endogenous expression of DLG1 and CDK-2 was ascertained using the specific anti-DLG1 or anti-CDK-2 antibodies. When indicated, CX-4945 CKII inhibitor was added to the cells 12h before harvesting. α-actinin was used as loading control. **Right panel**, densitometry analysis of western blots for DLG1 and CDK-2 expression, normalised for α-actinin levels in each corresponding different condition (mean ± SD, n = 3). Asterisks denote significant differences determined by ANOVA test (* $p<0.05$ and ** $p<0.005$). Original blots are presented in the Supplementary Figures 8

Figure Supplementary 1.

B

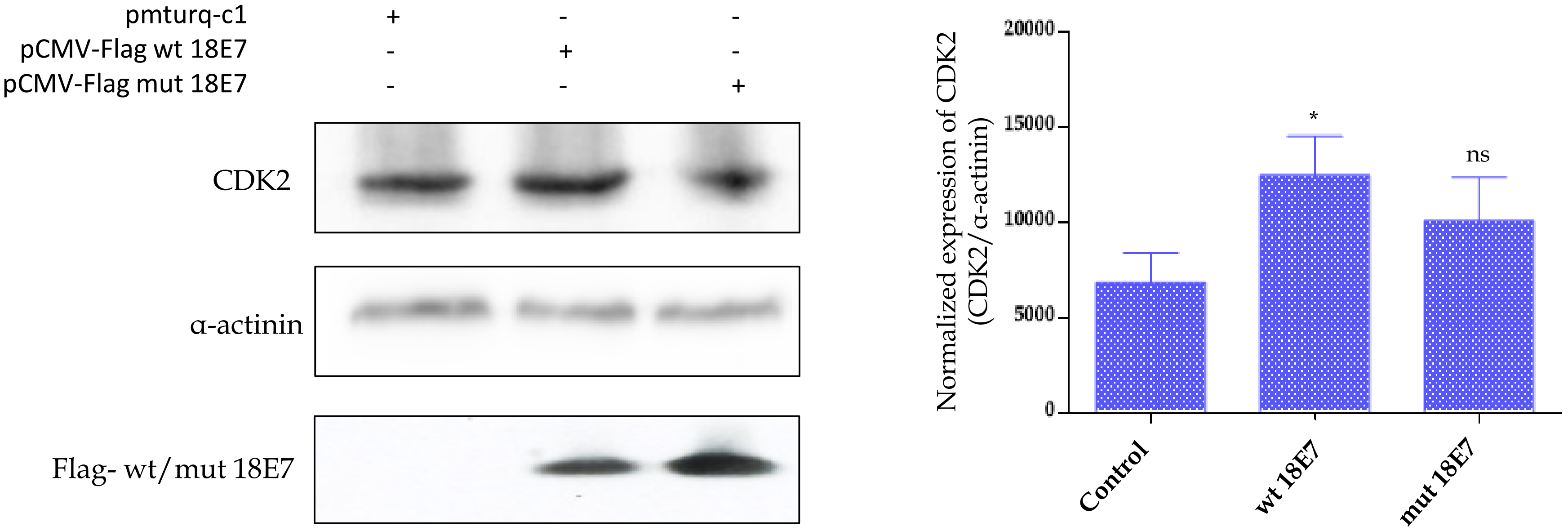

Fig S1 B. HPV-18 E7 protein increases the levels of CDK-2 in a CKII-dependent manner. HEK293 epithelial cells were transiently transfected with 5µg wild type or mutant pCMV-Flag-18 E7. Cells were harvested 24 h post-transfection and the expression of CDK 2 kinase was assessed by Western Blot (**Left panel**) using an anti-CDK-2 antibody. α-actin was used as loading control. **Right panel,** densitometry analysis of western blots for CDK-2 expression for equal α-actin levels in each corresponding different condition (mean ± SD, n = 3). Asterisks denote significant difference determined by ANOVA test and a Multiple Comparisons Tukey´s test (*p < 0.5,) and "ns" indicates no significant changes comparing with the control. Original blots are presented in the Supplementary Figures 9.

Figure Supplementary 2.

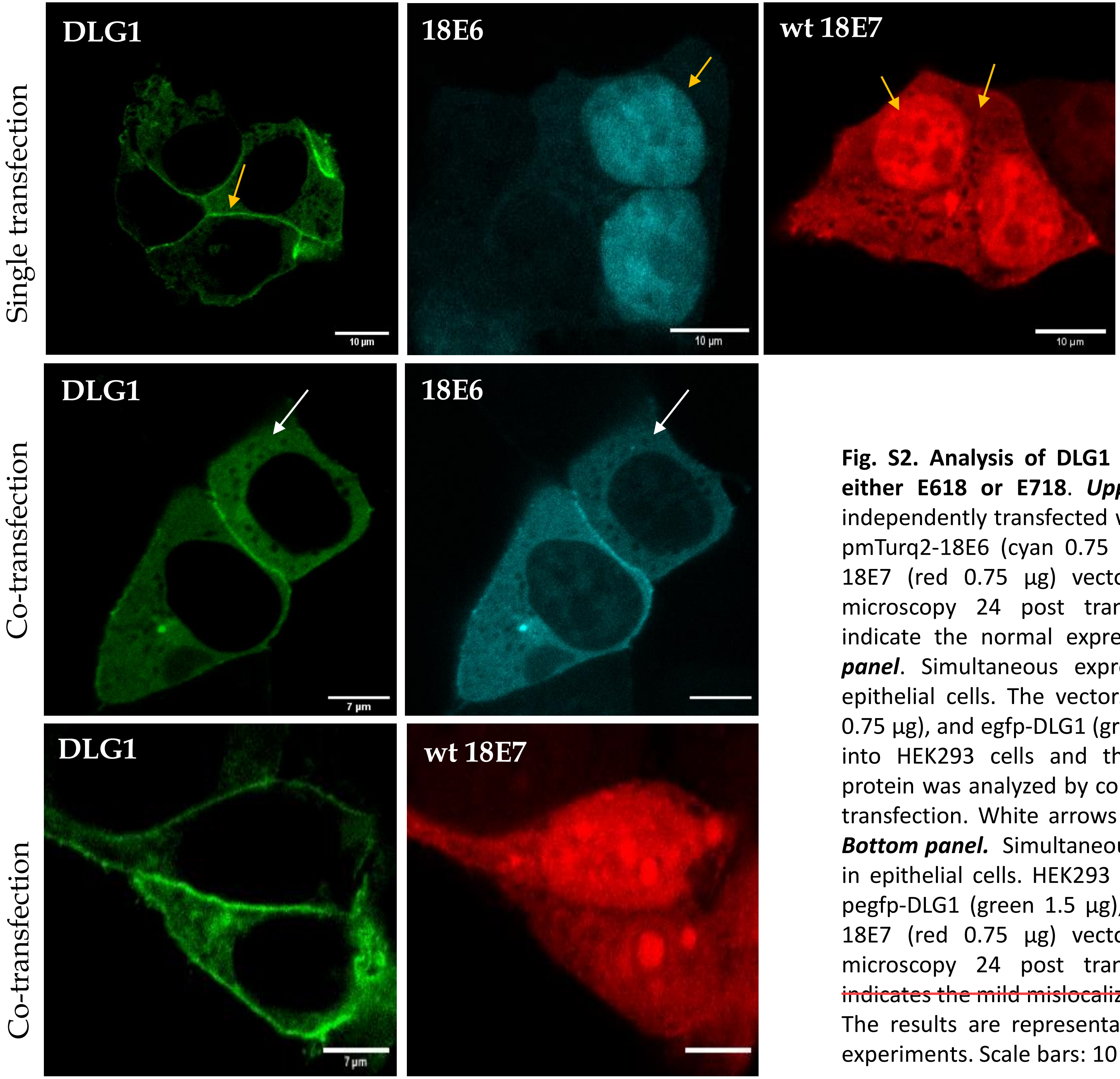

Fig. S2. Analysis of DLG1 expression in the presence of either E618 or E718. ***Upper panel***. HEK293 cells were independently transfected with pegfp-DLG1 (green 1.5 µg), pmTurq2-18E6 (cyan 0.75 µg), or wild type pLPC-Cherry-18E7 (red 0.75 µg) vectors and analysed by confocal microscopy 24 post transfection. The yellow arrows indicate the normal expression of the proteins. ***Middle panel***. Simultaneous expression of E618 and DLG1 in epithelial cells. The vectors encoding mTurq2-18E6 (cyan 0.75 µg), and egfp-DLG1 (green 1.5 µg) were co-transfected into HEK293 cells and the localization of each fusion protein was analyzed by confocal microscopy after 24 post transfection. White arrows indicate protein redistribution. ***Bottom panel.*** Simultaneous expression of E718 and DLG1 in epithelial cells. HEK293 cells were co- transfected with pegfp-DLG1 (green 1.5 µg), and wild type or pLPCCherry-18E7 (red 0.75 µg) vectors and analysed by confocal microscopy 24 post transfection. ~~The white arrow indicates the mild mislocalization of DLG1.~~

The results are representative of at least 4 independent experiments. Scale bars: 10 µm and 7 µm.

Figure Supplementary 3. Uncropped Figure 1 A.

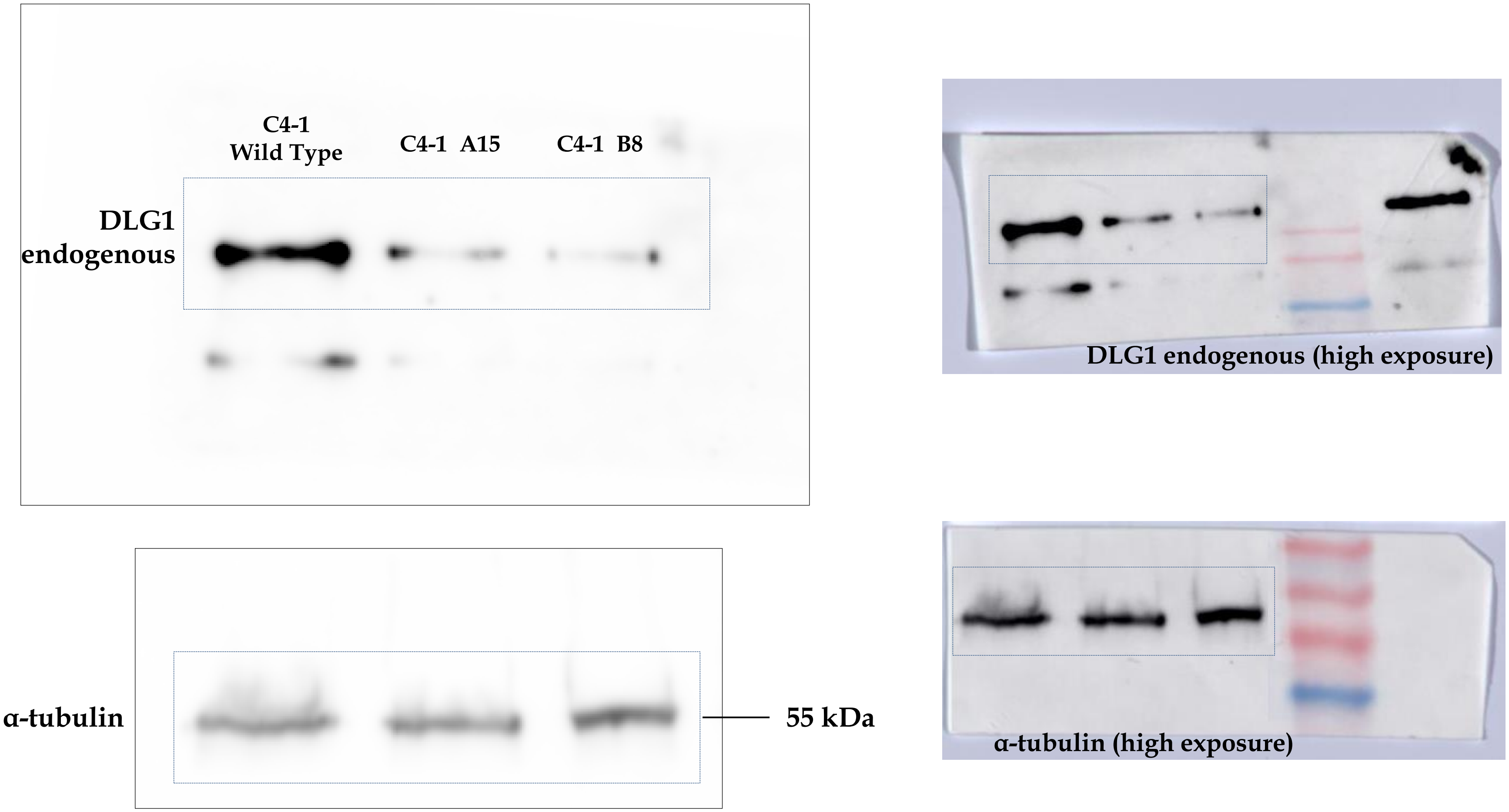

Figure Supplementary 4. Uncropped Figure 1 B.

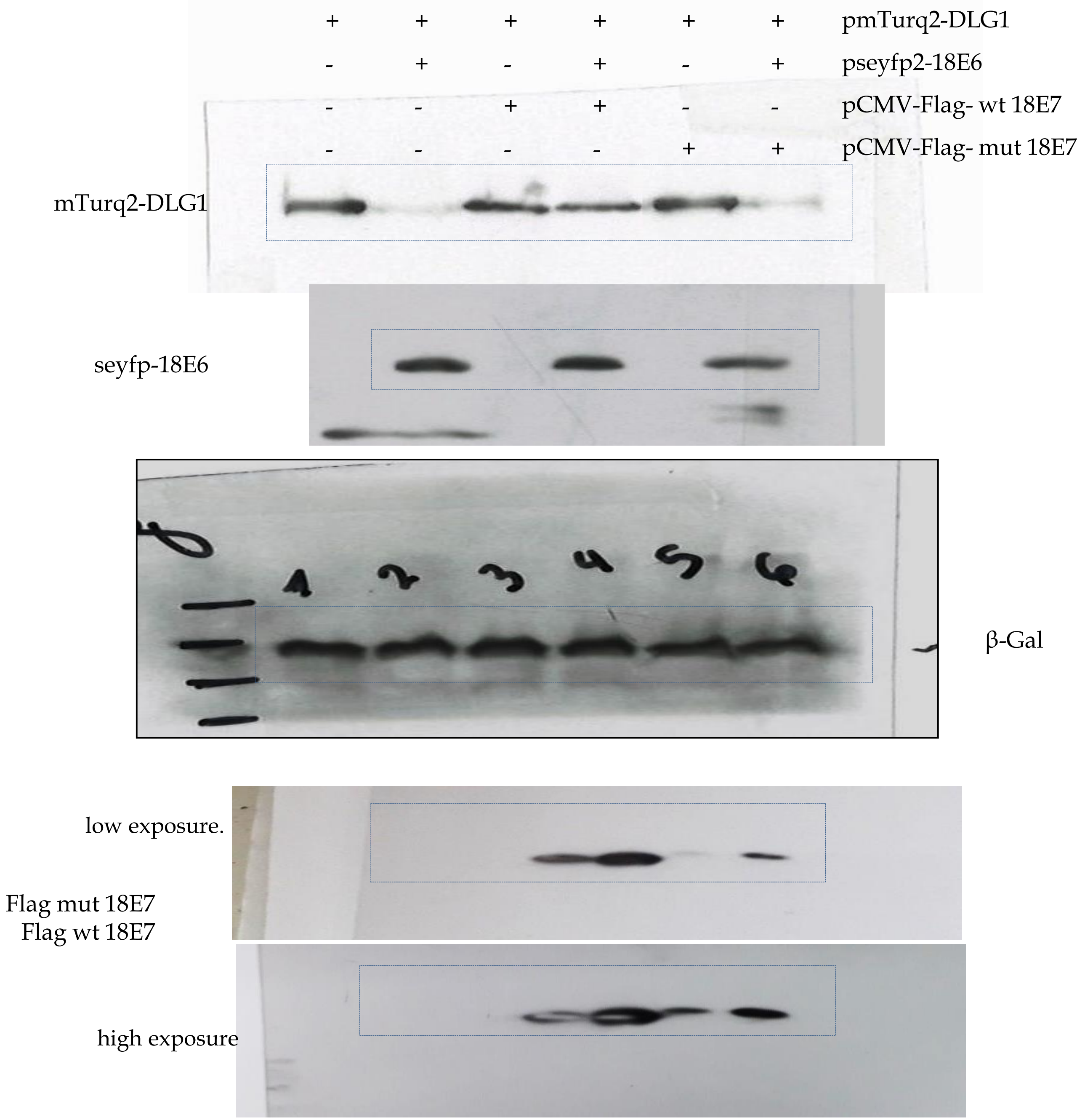

Figure Supplementary 5. Uncropped Figure 2.

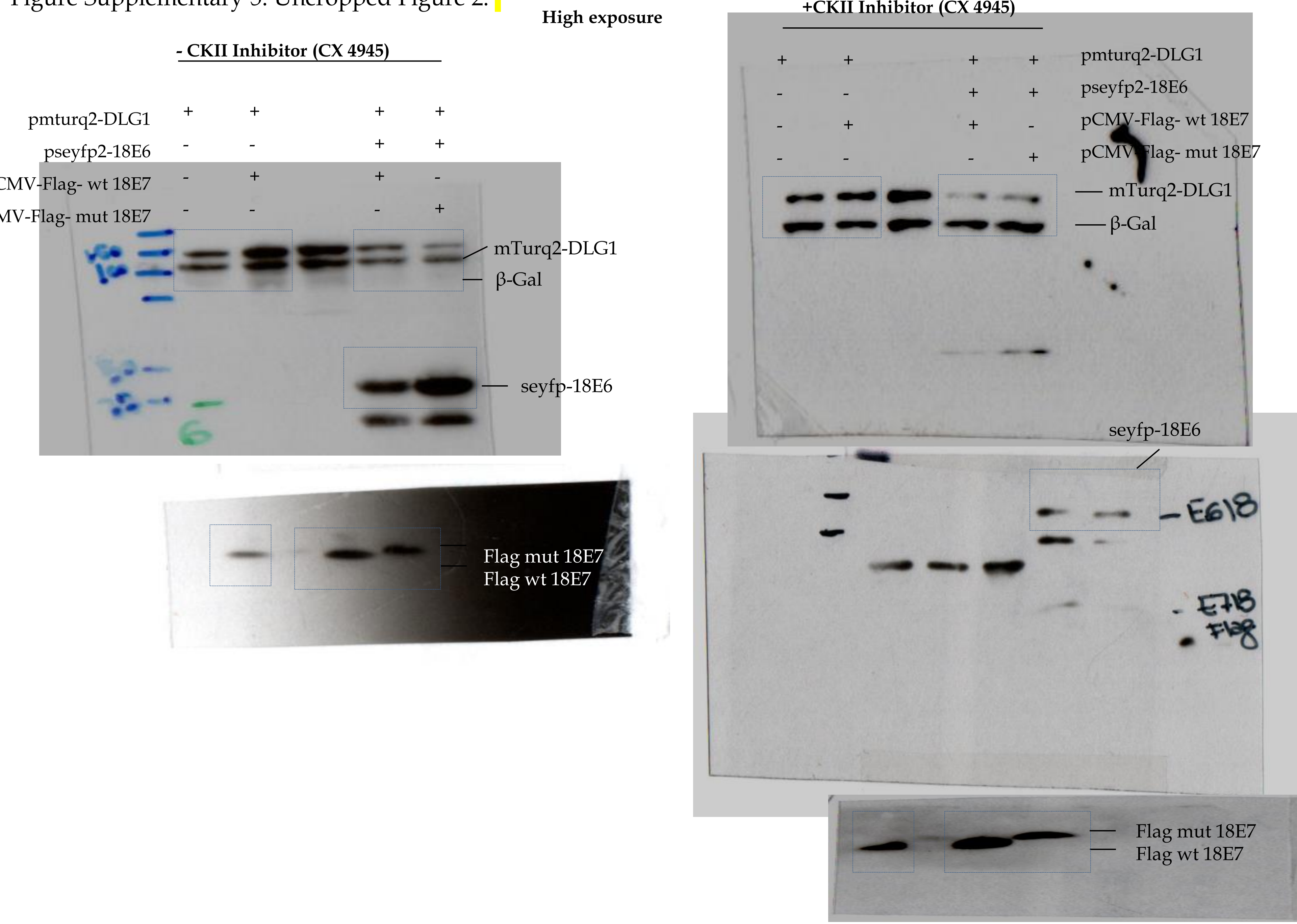

Figure Supplementary 5 (continuation). Uncropped Figure 2.

Low exposure.

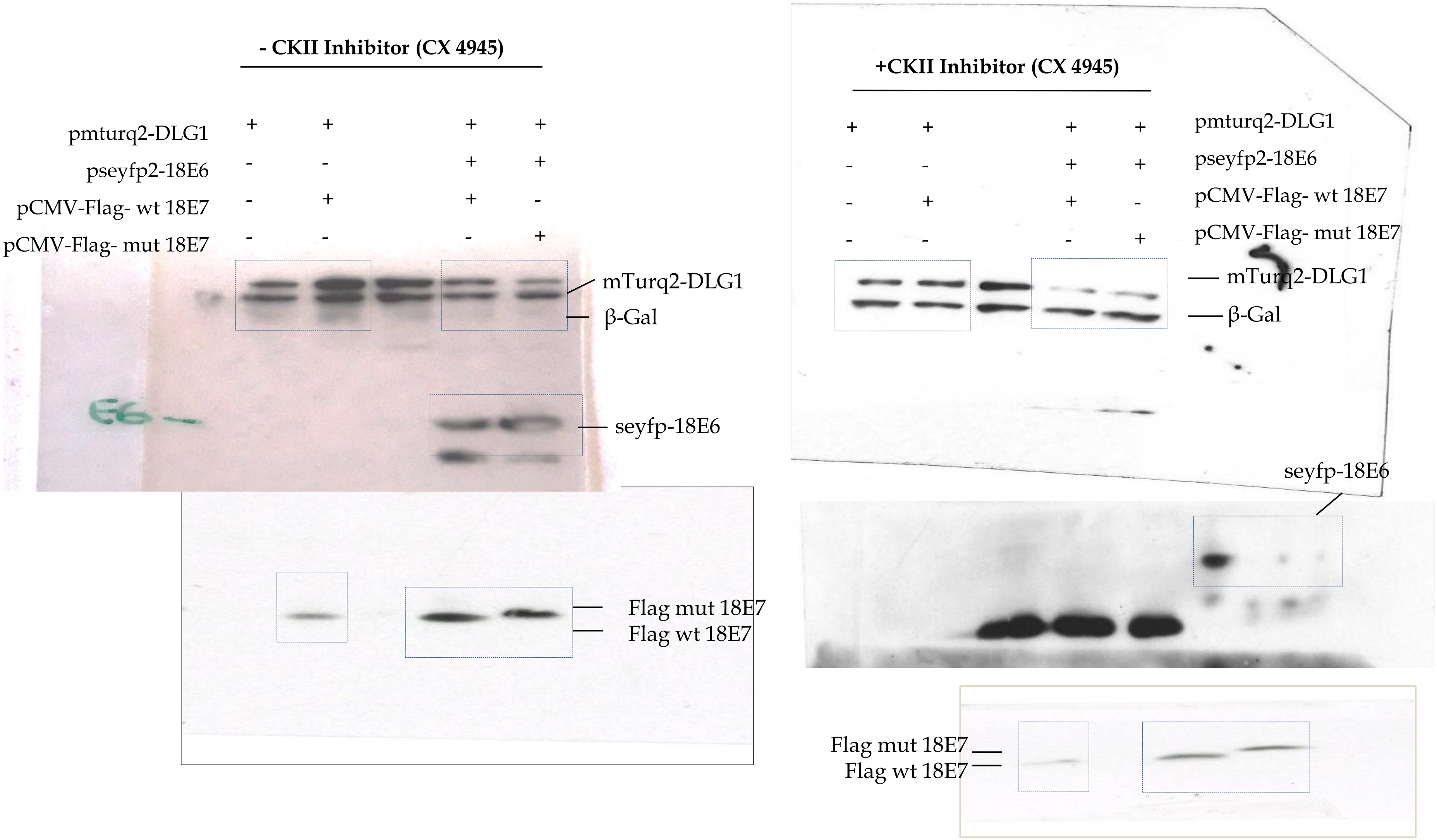

Figure Supplementary 6. Uncropped Figure 4 A.

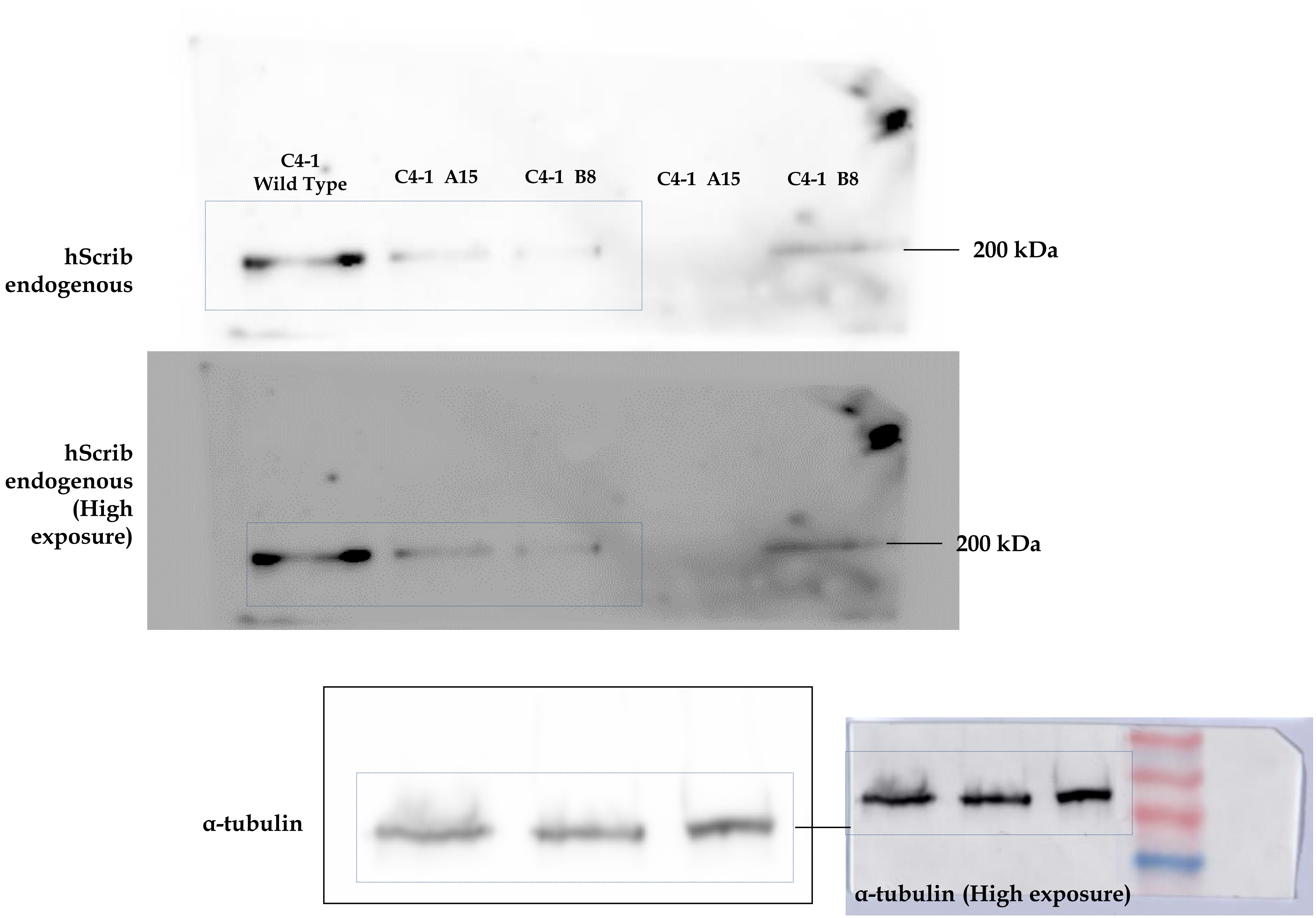

Figure Supplementary 7. Uncropped Figure 4 B.

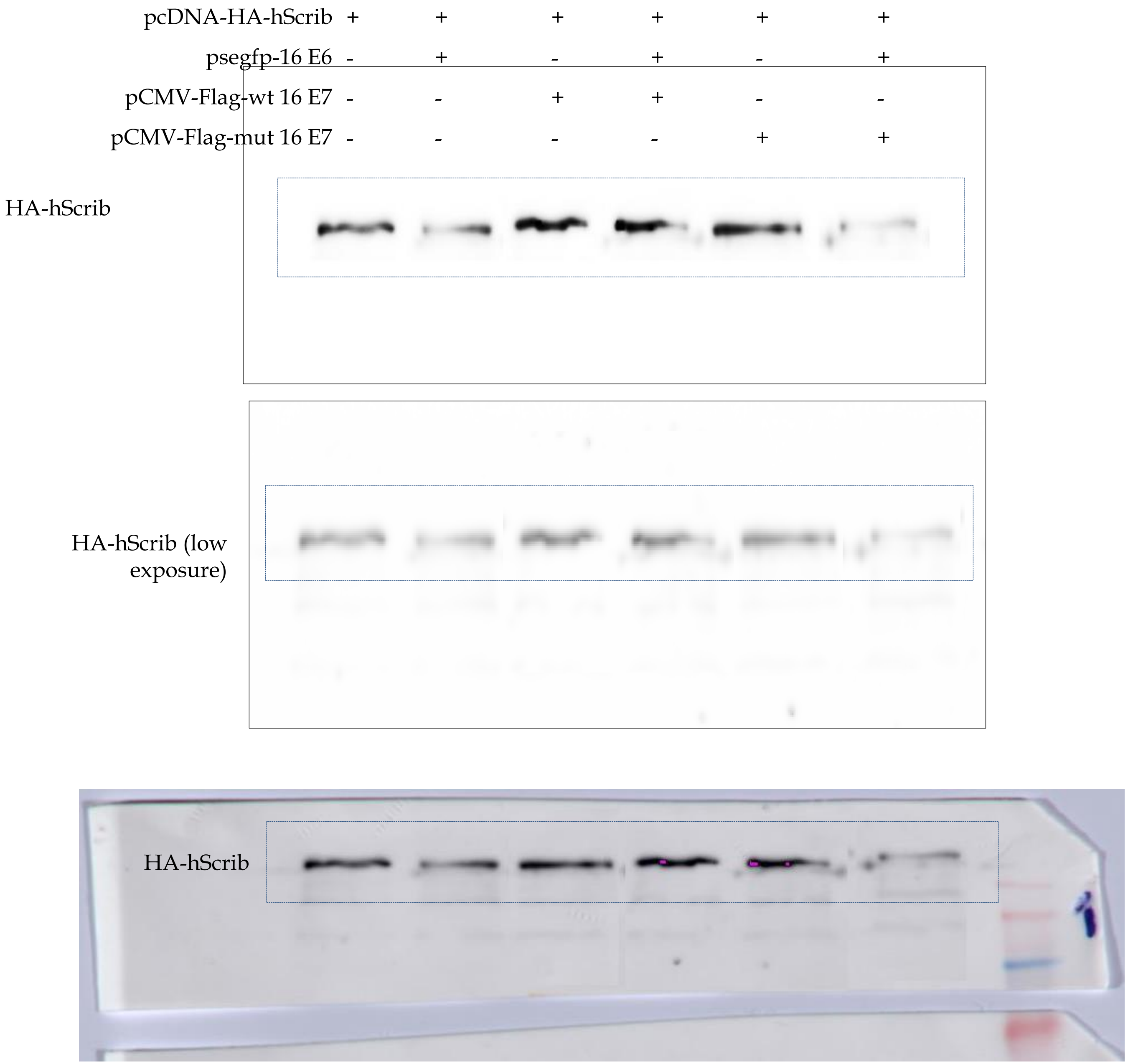

Figure Supplementary 7 (continuation). Uncropped Figure 4 B.

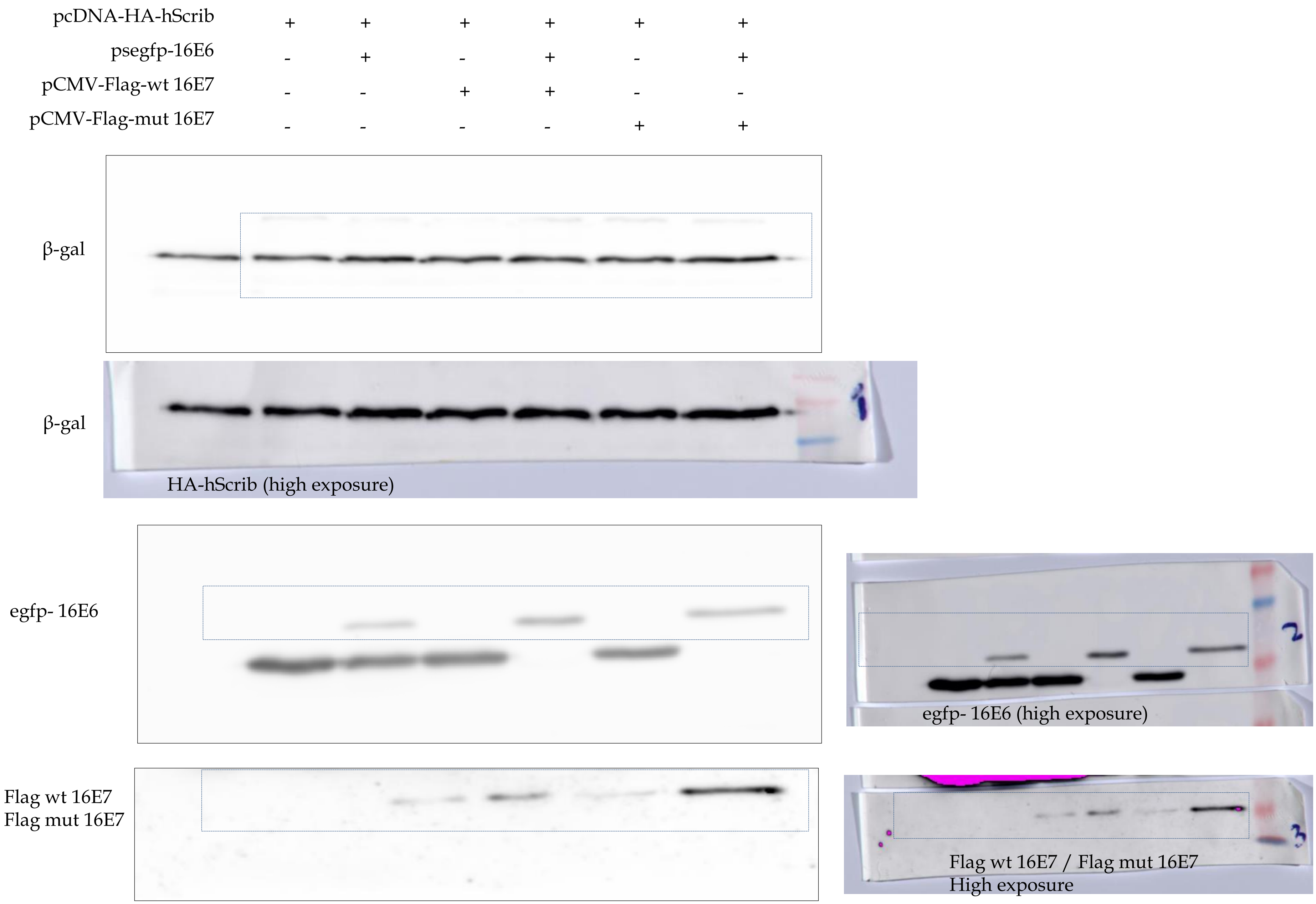

Figure Supplementary 8. Uncropped Figure Supplementary 1.

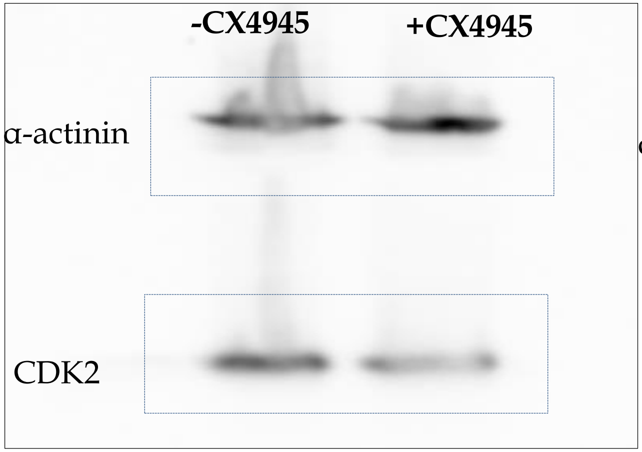

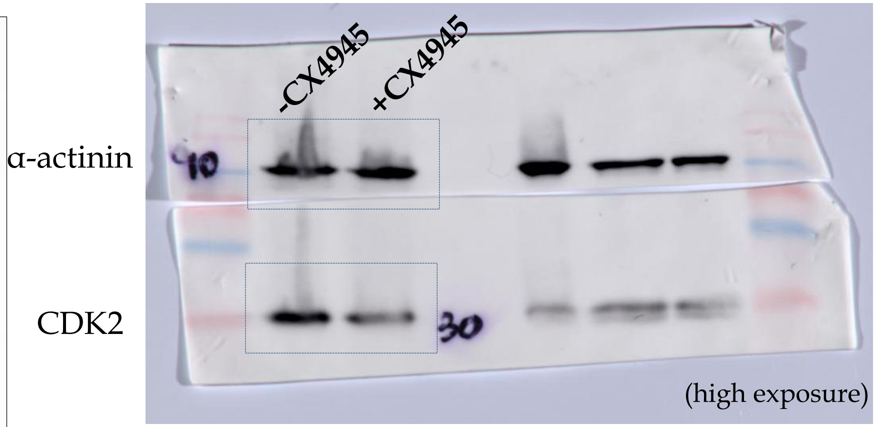

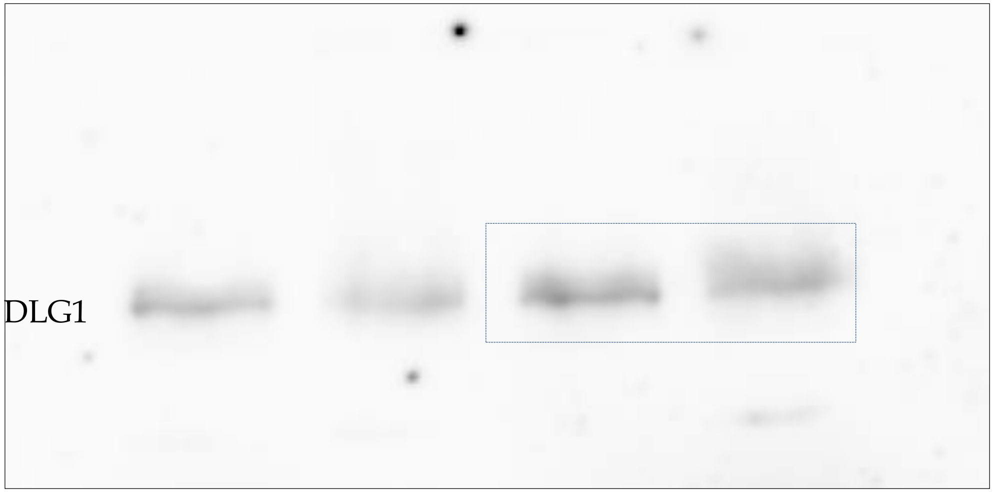

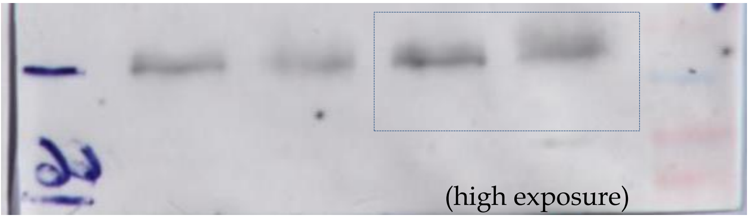

Figure Supplementary 9. Uncropped Figure Supplementary 1 B.

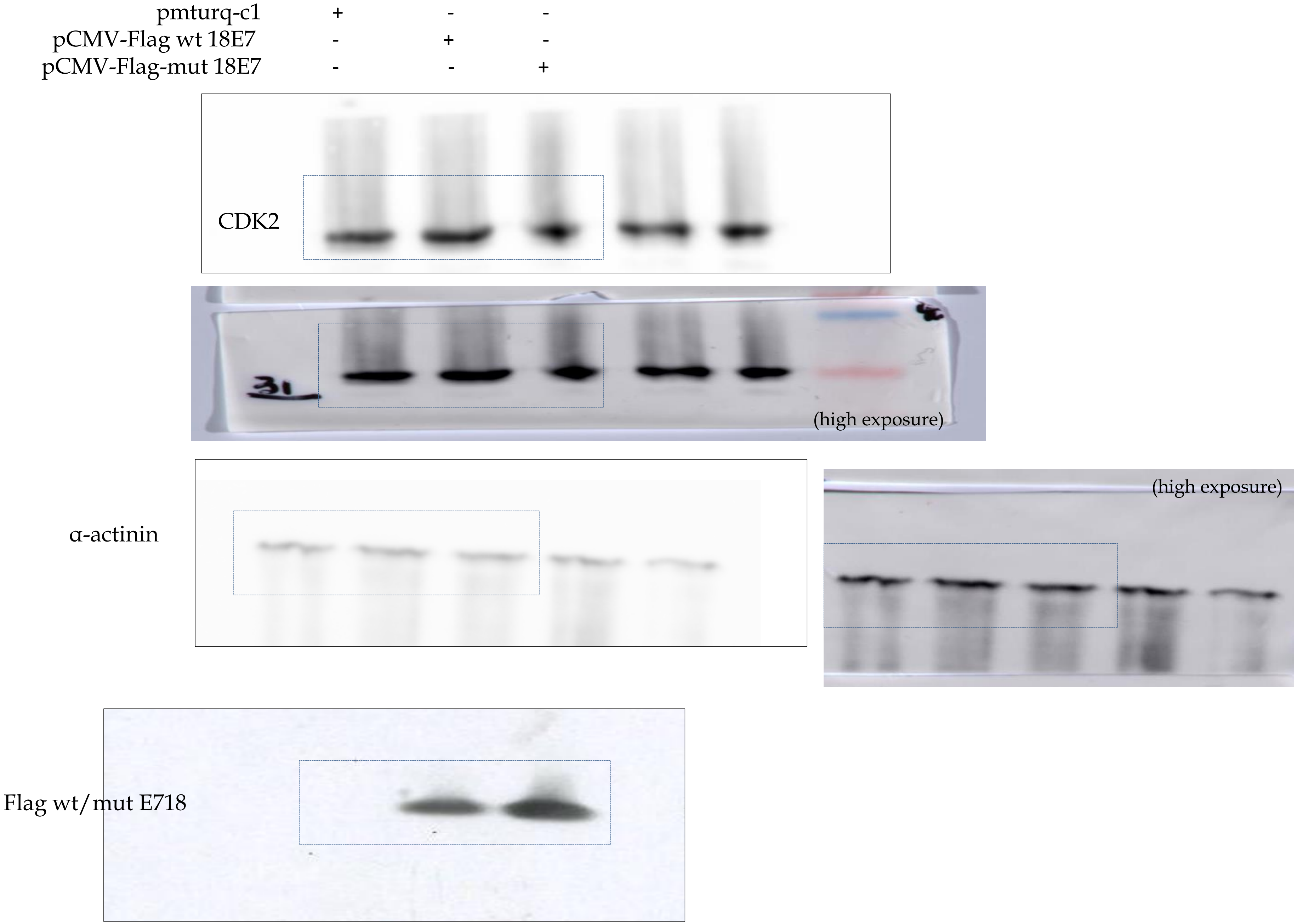

Supplement: Supplementary file 1 — Additional file 1: Fig. S1. A. CKII inhibitor induces a reduction in DLG1 and CDK-2 levels in HPV-positive cells. Fig. S1. B. HPV-18 E7 protein increases the levels of CDK-2 in a CKII-dependent manner. Fig. S2. Analysis of DLG1 expression in the presence of either E618 or E718. Upper panel. Figure Supplementary 3. UncroppedFigure 1 A. Figure Supplementary 4. Uncropped Figure 1 B. Figure Supplementary 5. Uncropped Figure 2. Figure Supplementary 6. UncroppedFigure 4 A. Figure Supplementary 7. Uncropped Figure 4 B. Figure Supplementary 8. Uncropped Figure Supplementary 1. Figure Supplementary 9. Uncropped Figure Supplementary 1 B. [file 12885_2022_10105_MOESM1_ESM.pdf]
